# Neuronal Sorbs2 Regulates Learning And Memory Through Multiple Mechanisms

**DOI:** 10.1002/alz70855_107563

**Published:** 2025-12-27

**Authors:** Grace Rocco, Henry Lin, Kailey A Said, Ryan L Boudreau, Jared M McLendon

**Affiliations:** ^1^ University of Iowa, Iowa City, IA, USA

## Abstract

**Background:**

Sorbs2 is a cytoskeletal adaptor protein that is expressed in hippocampal neurons, with genetic variants associated with an 11‐year delay in Alzheimer's Disease (AD) onset, but its mechanistic role in these cells is not yet fully understood.

**Method:**

We generated whole body Sorbs2‐Knockout (KO) mice and tested WT and KO littermates (*n* = 20 per genotype) using behavior tests; spatial object recognition [SOR] and cued fear conditioning [CFC]. We performed microtubule stability assays and synaptosome protein extractions to test molecular mechanisms of neuron dysfunction. We generated custom antibodies and RNAscope probes to detect different isoforms of Sorbs2 proteins and RNA, in specific cell types and brain regions. We performed RNA sequencing on Sorbs2‐WT and KO hippocampus to detect the novel role of Sorbs2 as a neuron specific RNA binding protein.

**Result:**

Sorbs2 KO mice interacted less with displaced objects in SOR tests and had reduced freezing behavior in CFC test, both suggesting impaired learning or memory. Synaptosome fraction assays show significant enrichment of Sorbs2 protein hippocampus (∼ 2‐fold) and neuronal and post synaptic density proteins (NeuN and Psd95) were also enriched in synaptosomes, however, not significantly different in KO mice. However, total expression of NeuN was significantly increased in KO mice suggesting altered neurogenesis in KO mice. RNA sequencing shows dysregulation of pathways related to microtubule and other cytoskeletal structures, however, in the hippocampus, there was also a upregulation of pathways related to ribosome assembly and translation initiation in KO mice. Next, we interrogated alternative splicing and found 6 genes with dysregulated isoform switching in Sorbs2‐KO hippocampus without changing the overall gene expression. Of note, the alternative splicing in each of these examples changes the 5’UTR and/or translation start site. Altogether, this suggests a novel RBP role for Sorbs2 in neurons, through regulating translation initiation.

**Conclusion:**

Our results suggest Sorbs2 regulates MT stability and alternative splicing in neurons and is involved in memory formation or retrieval. We have generated custom antibodies and RNAscope probes that identify Sorbs2‐expressing cell populations in the brain, investigate Sorbs2 isoform expression, and assess the intersection between Sorbs2 and AD phenotypes in mouse models of neurodegeneration.

